# Baseline diabetes as a way to predict CV outcomes in a lipid-modifying trial: a meta-analysis of 330,376 patients from 47 landmark studies

**DOI:** 10.1186/s12933-015-0226-z

**Published:** 2015-05-21

**Authors:** Michel P. Hermans, Evariste Bouenizabila, Daniel K. Amoussou-guenou, Sylvie A. Ahn, Michel F. Rousseau

**Affiliations:** Division of Endocrinology & Nutrition, Cliniques universitaires St-Luc and Institut de Recherche Expérimentale et Clinique (IREC), Université catholique de Louvain, Brussels, Belgium; Service de Maladies Métaboliques et Endocriniennes, Centre Hospitalier et Universitaire de Brazzaville, Brazzaville, Congo; Service d’Endocrinologie et Métabolisme, CNHU HKM Cotonou, Université d’Abomey-Calavi, Abomey-Calavi, Bénin; Division of Cardiology, Cliniques universitaires St-Luc and Pôle de Recherche Cardiovasculaire, Institut de Recherche Expérimentale et Clinique (IREC), Université catholique de Louvain, Brussels, Belgium

**Keywords:** Diabetes, Cardiovascular, Coronary heart disease, Clinical trial, Residual risk, Lipids

## Abstract

**Background:**

Diabetes is a major cardiovascular risk factor. However, its influence on the rate of occurrence of cardiovascular (CV) events during a clinical trial that included a diabetes subgroup has not yet been quantified.

**Aims:**

To establish equations relating baseline diabetes prevalence and incident CV events, based on comparator arms data of major lipid-modifying trials.

**Methods:**

Meta-analysis of primary outcomes (PO) rates of key prospective trials, for which the baseline proportion of diabetics was reported, including studies having specifically reported CV outcomes within their diabetic subgroups.

**Results:**

47 studies, representing 330,376 patients (among whom 124,115 diabetics), were analyzed as regards the relationship between CV outcomes rates (including CHD) and the number of diabetics enrolled. Altogether, a total of 18,445 and 16,156 events occurred in the comparator and treatment arms, respectively. There were significant linear relationships between diabetes prevalence and both PO and CHD rates (%/year): y = 0.0299*x + 3.12 [PO] (p = 0.0128); and y = 0.0531*x + 1.54 [CHD] (p = 0.0094), baseline diabetes predicting PO rates between 3.12 %/year (no diabetic included) and 6.11 %/year (all patients diabetic); and CHD rates between 1.54 %/year (no diabetic) and 6.85 %/year (all patients diabetic). The slopes of the equations did not differ according to whether they were derived from primary or secondary prevention trials.

**Conclusions:**

Absolute and relative CV risk associated with diabetes at inclusion can be readily predicted using linear equations relating diabetes prevalence to primary outcomes or CHD rates.

## Introduction

Key prospective trials have demonstrated the effectiveness of long-term control of conventional risk factors (RFs) to prevent cardiovascular (CV) events. Next to decreasing tobacco use and physical inactivity, indisputable gains were achieved by targeting hypertension and hypercholesterolemia. Nevertheless, there remained a high residual risk of incident CV events in control and comparator arms of these trials, even in patients receiving appropriate standard of care [1–4]. This residual risk is driven by non-modifiable RFs (age; gender; familial or genetic features; and diabetes) and by modifiable conventional or emerging RFs (eg. atherogenic dyslipidemia; remnant lipoproteins; hyperglycaemia; hyperinsulinaemia; metabolic syndrome; subclinical inflammation; and chronic kidney disease).

Based on epidemiology and prospective studies, type 2 diabetes mellitus (T2DM) significantly increases the absolute risk of developing coronary heart disease (CHD), and confers a higher residual risk of large and small vessel damage. In the microcirculation, such risk is directly related to hyperglycaemia, whereas in large vessels, this residual risk is linked to hypertension, low-density lipoproteins (LDL); non-LDL dyslipidemias; and other metabolic comorbidities [5–10]. As a result, having T2DM, either individually or at a sub-group level (within a cohort or population) increases residual CV risk to an extent that needs to be determined. Since residual risk varies considerably from one study to another, such an evaluation would require going beyond comparing CV outcomes rates in diabetic *vs.* nondiabetic subgroups of individual trials.

The aim of this work was to establish equations relating baseline diabetes prevalence and incident CV events, based on comparator arms data of major clinical trials having investigated the potential CV benefit of various pharmacological or dietary interventions targeting, in the vast majority, lipids and lipoproteins. We performed a systematic meta-analysis of CV outcomes rates of those key prospective studies, for which the baseline proportion of diabetics was reported and, where available, studies having reported CV outcomes of diabetic subgroups [11–90] (Table 1).

**Table 1:** Overview of 47 landmark prospective clinical trials with CV outcomes having included a substantial number and/or proportion of diabetic patients at baseline

	CV prevention	Patients	Diabetes		Active arm	Comparator arm	Follow-up	Publication year	Reference
		*n*	*n*	%	*n*	*n*	years		
4D	PP-SP	1255	1255	100	619	636	4.0	2005	[11]
4S	SP	4444	202	5	2221	2223	5.4	1994	[12–14]
diabetes substudy	SP	202	202	100	105	97	5.4	1997	[14]
ACCORD-Lipid	PP-SP	5518	5518	100	2765	2753	4.7	2010	[15, 16]
ADDITION-Europe	PP-SP	3055	3055	100	1678	1377	5.3	2011	[17, 18]
AFCAPS/TexCAPS	PP	6605	155	2	3304	3301	5.2	1998	[19, 20]
AIM-HIGH	SP	3414	1158	34	1718	1696	3.0	2011	[21, 22]
AleCardio	SP	7226	7226	100	3616	3610	2.0	2014	[23, 24]
ALERT	PP-SP	2102	396	19	1050	1052	5.1	2003	[25]
ALLHAT-LLT	PP-SP	10355	3638	35	5170	5185	4.8	2002	[26]
Alpha-Omega	SP	4837	1754	36	2404	2433	3.4	2010	[27]
ASCOT-LLA	PP	10305	2532	25	5168	5137	3.3	2003	[28, 29]
diabetes substudy	PP	2532	2532	100	1258	1274	3.3	2005	[29]
ASPEN	PP	2410	2410	100	1211	1199	4.0	2006	[30]
AURORA	PP-SP	2773	731	26	1389	1384	3.8	2009	[31, 32]
diabetes substudy	PP-SP	731	731	100	388	343	2.8	2011	[32]
BIP	SP	3090	309	10	1548	1542	6.2	2000	[33, 34]
CARDS	PP	2838	2838	100	1428	1410	3.9	2004	[35]
CARE	SP	4159	586	14	2081	2078	5.0	1998	[36–38]
diabetes substudy	SP	586	586	100	282	304	5.0	1998	[38]
CDP (clofibrate)	SP	3892	1517	39	1103	2789	6.2	1975	[39, 40]
CDP (niacin)	SP	3908	1524	39	1119	2789	6.2	1975	[39, 40]
dal-OUTCOMES	SP	15871	3882	24	7938	7933	2.6	2012	[41, 42]
DIS	PP	761	761	100	379	382	5.0	1991	[43]
FIELD	PP-SP	9795	9795	100	4895	4900	5.0	2005	[44–46]
GISSI-Prevenzione	SP	4271	582	14	2138	2133	2.0	2000	[47]
GREACE	SP	1600	313	20	880	720	3.0	2002	[48, 49]
diabetes substudy	SP	313	313	100	161	152	3.0	2003	[49]
HATS	SP	107	17	16	73	34	3.0	2001	[50]
HHS	PP	4081	108	3	2051	2030	5.0	1987	[51, 52]
diabetes substudy	PP	135	135	100	59	76	5.0	1992	[52]
HPS - MRC/BHF	PP-SP	20536	5963	29	10269	10267	5.0	2002	[53, 54]
diabetes substudy	PP-SP	5963	5963	100	2978	2985	4.8	2003	[54]
HPS2-THRIVE	SP	25673	8299	32	12838	12835	3.9	2013	[55]
IDEAL	SP	8888	1057	12	4439	4449	4.8	2005	[56, 57]
ILLUMINATE	PP-SP	15067	6661	44.2	7533	7534	1.0	2007	[58]
JELIS	PP-SP	18645	3040	16.3	9326	9319	4.6	2007	[59]
LEADER	PP-SP	1568	268	17	783	785	4.6	2002	[60, 61]
LIPID	SP	9014	782	9	4512	4502	6.1	1998	[62–64]
LIPS	SP	1677	202	12	844	833	3.9	2002	[65]
MEGA	PP	7832	1632	21	3866	3966	5.3	2006	[66]
ORIGIN	PP-SP	12536	11081	88.4	6281	6255	6.2	2012	[67]
PERFORM	SP	19120	5299	27.7	9562	9558	2.4	2011	[68]
Post-CABG	SP	1351	116	9	676	675	7.5	2000	[69, 70]
PREDIMED	PP	7447	3614	49	4997	2450	4.5	2013	[71]
PROACTIVE	SP	5238	5238	100	2605	2633	2.9	2005	[72, 73]
PROFIT-J	PP-SP	481	481	100	234	247	1.8	2014	[74]
PROSPER	PP-SP	5804	623	11	2891	2913	3.2	2002	[75]
RPS	PP-SP	12505	7494	60	6239	6266	5.0	2013	[76, 77]
SHARP	PP-SP	9270	2094	23	4650	4620	4.9	2011	[78]
STABILITY	SP	15828	5351	34	7924	7904	3.7	2014	[79, 80]
STENO-2	PP-SP	160	160	100	80	80	13.3	2008	[81]
TNT	SP	10001	1501	15	4995	5006	4.9	2005	[82–86]
diabetes substudy	SP	1501	1501	100	753	748	4.9	2006	[86]
VA Cooperative Study	SP	532	128	24	268	264	1.8	1973	[87]
VA-HIT	SP	2531	769	30	1264	1267	5.1	1999	[88–90]
diabetes substudy	SP	769	769	100	377	392	5.1	2002	[90]
Total (n)		330376	124115		165022	165354			
Mean							4.4		

CV: cardiovascular; PP and SP: primary and secondary prevention. Acronyms: 4D: Die Deutsche Diabetes Dialyse studie; 4S: Scandinavian Simvastatin Survival Study; ACCORD-Lipid: Action to Control Cardiovascular Risk in Diabetes - Lipid arm; ADDITION-Europe: Anglo-Danish-Dutch Study of Intensive Treatment in People with Screen Detected Diabetes in Primary Care; AFCAPS/TexCAPS: Air Force/Texas Coronary Atherosclerosis Prevention Study; AIM-HIGH: Atherothrombosis Intervention in Metabolic Syndrome with Low HDL/High Triglycerides: Impact on Global Health Outcomes; AleCardio: A Safety and Efficacy Study to Evaluate the Potential of Aleglitazar to Reduce CV Risk in CHD Patients with a Recent ACS and T2DM; ALERT: Assessment of Lescol in Renal Transplantation; ALLHAT-LLT: Antihypertensive and Lipid-Lowering treatment to prevent Heart Attack Trial; ASCOT-LLA: Anglo-Scandinavian Cardiac Outcomes Trial - Lipid Lowering Arm; ASPEN: Atorvastatin as Prevention of CHD Endpoints in patients with Non-insulin dependent diabetes mellitus; AURORA: A Study to Evaluate the Use of Rosuvastatin in Subjects on Regular Hemodialysis: an Assessment of Survival and Cardiovascular Events; BIP: Bezafibrate Infarction Prevention; CARDS: Collaborative Atorvastatin Diabetes Study; CARE : Cholesterol and Recurrent Events; CDP: Coronary Drug Project; dal-OUTCOMES: Efficacy and safety of dalcetrapib in patients with recent acute coronary syndrome; DIS: Diabetes Intervention Study; FIELD: Fenofibrate Intervention and Event Lowering in Diabetes; GISSI-Prevenzione: Gruppo Italiano per lo Studio della Sopravvivenza nell’Infarto miocardico - Prevenzione; GREACE: Greek Atorvastatin and Coronary-heart-disease Evaluation; HATS: HDL-Atherosclerosis Treatment Study; HHS: Helsinki Heart Study; HPS - MRC/BHF: Medical Research Council and British Heart Foundation Heart Protection Study; HPS2-THRIVE: Heart Protection Study - Treatment of HDL to Reduce the Incidence of Vascular Events; IDEAL: Incremental Decrease in End Points Through Aggressive Lipid Lowering Trial; ILLUMINATE: Investigation of Lipid Level Management to Understand its Impact in Atherosclerosis Events; JELIS: Japan EPA Lipid Intervention Study; LEADER: Lower Extremity Arterial Disease Event Reduction; LIPID: Long-term Intervention with Pravastatin in Ischaemic Disease; LIPS: Lescol Intervention Prevention Study; MEGA: Primary Prevention of Cardiovascular Disease with Pravastatin in Japan; ORIGIN: Outcome Reduction with an Initial Glarigine Intervention; PERFORM: Prevention of cerebrovascular and cardiovascular Events of ischaemic origin with teRutroban in patients with a history oF ischaemic strOke or tRansient ischaeMic attack; Post-CABG (FU): Post Coronary Artery Bypass Graft Trial (follow-up); PREDIMED: Prevencion con Dieta Mediterranea; PROACTIVE: PROspective pioglitAzone Clinical Trial In macroVascular Events; PROFIT-J: PRimary preventiOn oF hIgh risk Type 2 diabetes in Japan; PROSPER: Prospective Study of Pravastatin in the Elderly at Risk; RPS: Risk and Prevention Study; SHARP: Study of Heart and Renal Protection; STABILITY: STabilization of Atherosclerotic plaque By Initiation of darapLadIb TherapY; STENO-2: STENO-2 Study; TNT: Treating to New Targets; VA Cooperative Study: Veteran Administration Cooperative Study of Atherosclerosis, Neurology Section; VA-HIT: Veterans Affairs High-Density Lipoprotein Intervention Trial

## Patients and methods

To be selected for inclusion, major clinical trials with CV outcomes had to meet three requirements: (*i*) the main purpose of the trial was to study the effect on CHD of a pharmacological or dietary intervention targeting lipids or lipoproteins, with CHD rates as sole primary outcome (PO), or with a major adverse CV event (MACE) composite PO comprising CHD; (*ii*) to focus exclusively on diabetic patients, or (*iii*) to report data on a sufficient number of diabetic patients from *pre-/post-hoc* analyses of DM subgroups of the main trial. Among studies conducted non-exclusively in DM patients, eligible trials had to comply with ≥1 of the following criteria: (*i*) the main trial had a subgroup of patients already diagnosed with DM at baseline, whose proportion was deemed sufficiently representative (>15 %); or (*ii*) the trial enrolled at least 100 DM patients, regardless of on-study new-onset diabetes.

For each study, the following items were analyzed: CV risk category at baseline (primary prevention [PP], secondary prevention [SP] or mixed [PP-SP]); number of patients included; number and proportion of patients with DM at baseline; number of patients in the active or comparator arms; duration of follow-up; age at inclusion; number of males; DM type and duration; HbA_1c_; total cholesterol (TC); low-density lipoprotein cholesterol (LDL-C); high-density lipoprotein cholesterol (HDL-C); non-HDL-cholesterol (non-HDL-C); apolipoprotein B_100_ (apoB); triglycerides (TG); type of pharmacological or dietary intervention; primary trial outcome; CHD outcomes (*see* Table 2*for CV outcomes categories*); and CV events number and rates for each trial.

**Table 2:** CV outcomes categories

Total mortality	all-cause death	A
Composite	all CV events (including procedures)	B
	MACE	C
	CV death	D
Cardiac	total CHD/major coronary events	E
	nonfatal CHD	F
	cardiac death/fatal CHD	G
	ACS/ACE	H
	all MI	I
	nonfatal MI	J
	fatal MI	K
	unstable/hospitalization-requiring AP	L
	coronary revascularization (PCI or CABG)	M
	life-threatening arrhytmias	N
	resuscitation for cardiac arrest	O
	sudden death	P
	CHF	Q
Coronary imaging	angiographic CAD progression/change in coronary atheroma volume	R
Cerebrovascular	all major cerebrovascular events	S
	all stroke/TIA	T
	nonfatal stroke	U
	fatal stroke	V
	carotid revascularization	W
Other composite	non-CHD MACE	X
Other mortality	non-CHD CV death	Y
Peripheral	any PAD event (including revascularization and leg amputation)	Z

ACE/ACS: acute coronary event/syndrome; AP angina pectoris; CABG: coronary artery bypass graft; CAD: coronary artery disease; CHD: coronary heart disease; CHF: congestive heart failure; CV: cardiovascular; MACE: major adverse cardiovascular event; MI myocardial infraction; PAD: peripheral arterial disease; PCI: percutaneous coronary intervention; TIA:transient is chemic attack (adapted from [91])

Results are presented as means (±1 standard deviation (SD)), or as proportions (%), with between-study range [BSR] described when needed. Linear regression was computed using the least-squares method. Results were considered statistically significant or non-significant (NS) for p <0.05 or p ≥0.05, respectively.

## Results

Forty-seven studies were selected based on the criteria defined above [11–90]. They accounted for a total of 330,376 patients. The median year of publication for all studies was 2005. Table 1 describes, for each study, the acronym’s definition; the CV prevention category; the cohort size and the number or proportion of diabetic at baseline; the number of patients randomized in the active or comparator arms; the follow-up duration; and publication year. For all studies, mean age (1SD) was 61.7 (6.4) years, and the proportion of males was 74 (17) %. Regarding ethnicity, the majority of patients studied were Caucasian (median 86.5 % [between-study range (BSR 0 %)–99.2 %] Three studies [JELIS; MEGA; and PROFIT-J] included only Japanese patients [59, 66, 74]. Among studies, 8 of 47 (17 %; *n* = 42,279) enrolled patients in PP at baseline; 17 of 47 (36 %; *n* = 131,425) included populations whose CV risk was a mix of PP and SP; and 22 of 47 (47 %; *n* = 156,672) were SP trials. Lipid values at baseline were (mg/dL): 209 (34) [TC]; 126 (32) [LDL-C]; 44 (7) [HDL-C]; 161 (32) [non-HDL-C]; 99 (19) [apoB] and 162 (27) [TG]. In total, these studies have included 124,115 diabetic patients, representing 42.1 % [BSR 2.3 %–100 %] of the population studied. For studies that reported diabetes duration, it averaged 7.5 (4.9) years, whereas metabolic control assessed by HbA_1c_ was 7.49 (0.68) % (Table 3). The trials investigated the following interventions over a mean (1SD) duration of 4.4 (1.9) years [BSR: 1.0–13.3 years]: statins (21 trials); fibrates (9 trials); n-3 fatty acids and/or traditional Mediterranean diet (5 trials); niacin (4 trials); CETP-inhibitor (2 trials); PPAR-γ agonist (2 trials); ezetimibe (1 trial); PPAR-α/γ agonist (1 trial); and Lp-PLA2 inhibitor (1 trial) (Table 4).

**Table 3:** Baseline characteristics

Study^§^	Age (years)	Males (%)	Diabetes type & duration (years)		HbA1c (%)	TC (mg/dL)	LDL-C (mg/dL)	HDL-C (mg/dL)	Non-HDL-C (mg/dL)	apoB (mg/dL)	TG (mg/dL)
4D	66	54	T2DM	18	6.7	218	125	36	182	~	261
4S	59	81	~			260	188	46	214	~	132
diabetes substudy	60	78	~			259	186	43	216	~	150
ACCORD-Lipid	62	69	T2DM	10	8.3	175	100	38	137	~	164
ADDITION-Europe	60.3	58	T2DM	0	7	214	133	46	168	~	146
AFCAPS/TexCAPS	58	85	T1DM; T2DM			221	150	37	184	~	158
AIM-HIGH	64	85	~		6.7	146	74	35	111	83	168
AleCardio	60.8	73	T2DM	8.6	7.8	152	79	42	110	~	152
ALERT	50	66	~			247	158	50	197	~	195
ALLHAT-LLT	66	51	T2DM			224	146	48	176	~	152
Alpha-Omega	69	78	~			183	100	50	133	~	146
ASCOT-LLA	63	81	~			212	131	50	162	~	150
diabetes substudy	63.6	76	T2DM			205	128	46	159	~	168
ASPEN	61	66	T2DM	8	7.8	194	113	47	147	~	147
AURORA	64	62	~			176	100	45	131	82	157
diabetes substudy	65	66	~			174	97	43	131	~	168
BIP	60	91	T2DM			212	148	35	177	~	145
CARDS	62	68	T2DM	8	7.9	207	117	54	153	117	173
CARE	59	86	~			209	139	39	170	~	156
diabetes substudy	61	80	~			206	136	38	168	~	164
CDP (clofibrate)		100	~			252	~	~	~	~	183
CDP (niacin)		100	~			253	~	~	~	~	183
dal-OUTCOMES	60.2	81	~			145	76	42	103	81	134
DIS	46	56	T2DM	0		218	~	~	~	~	157
FIELD	62	63	T2DM	5	6.9	195	119	43	152	97	173
GISSI-Prevenzione	60	86	T2DM (79 %) T1DM (21 %)			229	152	46	183	~	166
GREACE		79	~			264	193	39	225	~	159
diabetes substudy	55	56	T2DM (92 %) T1DM (8 %)	10.5	7.5	271	189	35	236	~	221
HATS	53	87	~			200	128	30	170	119	219
HHS	47	100	~			270	189	47	223	~	175
diabetes substudy	49	100	T2DM	4.5		292	200	46	246	~	214
HPS - MRC/BHF		75	~			228	131	41	187	114	186
diabetes substudy	62.1	70	T2DM (90 %) T1DM (10 %)	27	7	220	124	41	179	110	204
HPS2-THRIVE	64.9	82.7	~			128	63	44	84	68	127
IDEAL	62	81	~			197	122	46	151	119	151
ILLUMINATE	61.3	77.8	T2DM			157	80	49	108	73	127
JELIS	61	31.4	~			275	181	59	216	~	153
LEADER	68	100	~			218	131	46	172	~	213
LIPID	62	83	~			218	150	36	182	133	142
LIPS	60	84	T2DM; T1DM			200	131	38	162	~	160
MEGA	58.3	32	~			242	157	58	184	~	128
ORIGIN	63.5	65	T2DM	5.4		189	112	46	143	~	142
PERFORM	67.2	62.5	~			~	93	~	~		
Post-CABG	61.7	92	~			226	156	39	187	~	158
PREDIMED	67	43	~			219	143	53	172	102	142
PROACTIVE	61.8	66	T2DM	9.5	8.1	199	114	45	154	~	198
PROFIT-J	85	65	T2DM	11.3	7.4	198	115	55	144	~	141
PROSPER	75	48	~			220	147	50	170	~	133
RPS	63.9	61.5	~		6.7	216	132	51	165	~	150
SHARP	62	63	~			189	107	43	146	92	205
STABILITY	65	81	~			~	80	45	~		
STENO-2	54.9	74	T2DM	5.8	8.6	210	133	40	170	~	159
TNT	61	81	~			175	97	47	128	111	151
diabetes substudy	63	73	~	8.5	7.4	175	96	45	130	113	171
VA Cooperative Study	55	100	~			244	~	~	~	~	~
VA-HIT	64	100	~			175	111	32	143	96	161
diabetes substudy	65		~			172	108	31	141	~	166
mean	61.7	74		7.5	7.49	209	126	44	161	99	162
standard deviation	6.4	17		4.9	0.68	34	32	7	32	19	27

^§^: see legend to Table 1 for study acronyms definition; apoB: apolipoprotein B100; C: cholesterol; HbA1c: glycated haemoglobin A1c; HDL: high-density lipoprotein; LDL: low-density lipoprotein; T1DM and T2DM: type 1 and type 2 diabetes mellitus; TG: triglycerides

**Table 4:** Primary CV outcome rates in the active (treatment) and control (comparator/placebo) arms

Study^§^	Intervention	Primary; secondary CV outcomes^§§^	Events (n) treatment	Events (%) treatment	Rate (%.year-1) treatment	Events (n) control	Events (%) control	Rate (%.year-1) control	HR	95 % CI for HR	*P*
4D	statin	C; D + J	226	36.5	9.13	243	38.2	9.55	0.96	0.77-1.1	*0.37*
4S	statin	A	182	8.2	1.52	256	11.5	2.13	0.71	0.58-0.85	*0.0003*
diabetes substudy	statin	A	15	14.3	2.65	24	24.7	4.58	0.58	NR	*0.087*
ACCORD-Lipid	fibrate	C; J + D	291	10.5	2.24	310	11.3	2.40	0.93	0.79-1.08	*0.32*
ADDITION-Europe	statin/other	B; D + J + M + Z	121	7.2	1.36	117	8.5	1.60	0.85	0.65-1.05	*0.12*
AFCAPS/TexCAPS	statin	C; E	116	3.5	0.68	183	5.5	1.07	0.63	0.50-0.79	*<0.001*
AIM-HIGH	niacin	C; G + J + H + M	282	16.4	5.47	274	16.2	5.39	1.02	0.87-1.21	*0.8*
AleCardio	PPAR-α/γ	C; D + J	344	9.5	4.76	360	10.0	4.99	0.95	0.83-1.11	*0.57*
ALERT	statin	C; G + J + M	112	10.7	2.09	134	12.7	2.50	0.84	0.64-1.06	*0.14*
ALLHAT-LLT	statin	A	631	12.2	2.54	641	12.4	2.58	0.99	0.89-1.11	*0.88*
Alpha-Omega	n-3 fatty acids	B	336	14.0	4.11	335	13.8	4.05	1.02	0.87-1.17	*0.93*
ASCOT-LLA	statin	J + G	100	1.9	0.59	154	3.0	0.91	0.65	0.50-0.83	*0.0005*
diabetes substudy	statin	B	116	9.2	2.79	151	11.9	3.59	0.78	0.61-0.98	*0.04*
ASPEN	statin	C; D + J + M + O + L	166	13.7	3.43	180	15.0	3.75	0.91	0.73-1.12	*0.34*
AURORA	statin	C; J + D	396	28.5	7.50	408	29.5	7.76	0.97	0.84-1.11	*0.59*
diabetes substudy	statin	C; G + J	85	21.9	7.82	104	30.3	10.83	0.72	0.51-0.90	*0.008*
BIP	fibrate	C; K + J + P	211	13.6	2.20	232	15.0	2.43	0.91	NR	*0.26*
CARDS	statin	C; H + M + T	83	5.8	1.49	127	9.0	2.31	0.65	0.48-0.83	*0.001*
CARE	statin	G + J	212	10.2	2.04	274	13.2	2.64	0.77	0.09-0.36	*0.003*
diabetes substudy	statin	G + J + M	81	28.7	5.74	112	36.8	7.37	0.78	NR	*<0.0001*
CDP (clofibrate)	fibrate	A	281	25.5	4.11	709	25.4	4.10	1.00	NR	*NR*
CDP (niacin)	niacin	A	273	24.4	3.93	709	25.4	4.10	0.96	0.85-1.08	*NR*
dal-OUTCOMES	CETP inhibitor	C; G + J + L + O	656	8.3	3.20	633	8.0	3.09	1.04	0.93-1.16	*0.52*
DIS	fibrate	E	32	8.4	1.69	31	8.1	1.62	1.04	NR	*NR*
FIELD	fibrate	C; B + D + I + M	256	5.2	1.05	288	5.9	1.18	0.89	0.75-1.05	*0.16*
GISSI-Prevenzione	statin	C; A + I	120	5.6	2.77	136	6.4	3.15	0.88	0.71-1.15	*0.41*
GREACE	statin	C; A + J + L + Q + M	112	12.7	4.24	180	25.0	8.33	0.51		*<0.0001*
diabetes substudy	statin	C; A + J + L + Q + M	20	12.4	4.14	46	30.3	10.09	0.41	NR	*<0.0001*
HATS	statin + niacin^§§§^	R + B; D + J + M	7	9.6	3.20	12	35.3	11.76	0.27	NR	*0.02*
HHS	fibrate	C; K + J + G	56	2.7	0.55	84	4.1	0.83	0.66	0.08-0.53	*<0.02*
diabetes substudy	fibrate	C; K + J + G	2	3.4	0.68	8	10.5	2.11	0.32	NR	*0.19*
HPS - MRC/BHF	statin	C; A + G	1328	12.9	2.59	1507	14.7	2.94	0.88	0.81-0.94	*0.0003*
diabetes substudy	statin	E + B	601	20.2	4.20	748	25.1	5.22	0.81	0.19-0.30	*<0.0001*
HPS2-THRIVE	niacin	C; G + M	1696	13.2	3.39	1758	13.7	3.51	0.96	0.90-1.03	*0.29*
IDEAL	statin	C; G + J + O	411	9.3	1.93	463	10.4	2.17	0.89	0.78-1.01	*0.07*
ILLUMINATE	CETP inhibitor	C; G + J + L	464	6.2	6.16	373	5.0	4.95	1.24	1.09-1.44	*0.001*
JELIS	n-3 fatty acids	E; P; I; L; M; A	262	2.8	0.61	324	3.5	0.76	0.81	0.69-0.95	*0.01*
LEADER	fibrate	E	150	19.2	4.95	160	20.4	5.20	0.95	0.76-1.21	*0.72*
LIPID	statin	G	287	6.4	1.04	373	8.3	1.36	0.77	0.12-0.35	*<0.001*
LIPS	statin	C; G + J + M	181	21.4	5.50	222	26.7	6.83	0.80	0.64-0.95	*0.01*
MEGA	statin	C; I + L + M + P	66	1.7	0.32	101	2.5	0.48	0.67	0.49-0.91	*0.01*
ORIGIN	n-3 fatty acids	D; D + J + U; A; I; T; M + W; Q; L; Z	574	9.1	1.47	581	9.3	1.50	0.98	0.87-1.10	*0.72*
PERFORM	antiplatelet	D; I	1091	11.4	4.83	1062	11.1	4.71	1.03	0.94-1.12	*NS*
Post-CABG	statin	C; D + J + M	207	30.6	4.08	271	40.1	5.35	0.76	NR	*0.04*
PREDIMED	TMD	C; D + I	179	3.6	0.80	109	4.4	1.12	0.71		
PROACTIVE	glitazone	C; A + J + H + M	514	19.7	6.80	572	21.7	7.49	0.91	0.80-1.02	*0.1*
PROFIT-J	glitazone	C; A + J	9	3.8	2.09	10	4.0	2.20	0.95	0.427-2.593	*0.91*
PROSPER	statin	C; G + J	408	14.1	4.41	473	16.2	5.07	0.87	0.74-0.97	*0.01*
RPS	n-3 fatty acids	D	733	11.7	2.35	745	11.9	2.38	0.99	0.88-1.08	*0.64*
SHARP	statin/ezetimibe	C; J + G + M	526	11.3	2.31	619	13.4	2.73	0.84	0.74-0.94	*0.0021*
STABILITY	Lp-PLA2-inhibitor	C; D + J + U	769	9.7	2.62	819	10.4	2.80	0.94	0.85-1.03	*0.2*
STENO-2	statin/fibrate	A	24	30.0	2.26	40	50.0	3.76	0.60	0.32-0.89	*0.02*
TNT	statin	C; G + J + O + T	434	8.7	1.77	548	10.9	2.23	0.79	0.69-0.89	*<0.001*
diabetes substudy	statin	C; G + J + O + T	103	13.7	2.79	135	18.0	3.68	0.76	0.58-0.97	*0.026*
VA Cooperative Study	fibrate	A + B	22	8.2	4.56	30	11.4	6.31	0.72	0.43-1.22	*NR*
VA-HIT	fibrate	C; J + G	219	17.3	3.40	275	21.7	4.26	0.80	0.07-0.35	*0.006*
diabetes substudy	fibrate	C; J + G	96	25.5	4.99	141	36.0	7.05	0.71	0.53-0.88	*0.004*
Total (n)			16156			18445					
Mean				12.2	3.0		14.8	3.6	0.85		

^§^: see legend to Table 1 for study acronyms definition; §§: see Table 2 for CV outcomes definition; §§§: ±antioxidants; CETP: cholesteryl ester transfer protein; CI: confidence interval; CV: cardiovascular; HR: hazard ratio; LpPLA2: lipoprotein-associated phospholipase A2; NR: not reported; NS: non significant; PPAR: peroxisome proliferator-activated receptor; TMD: traditional Mediterranean diet

For all 47 studies, a total of 18,445 and 16,156 events occurred in the comparator and treatment arms, respectively. On an annual basis, this was equivalent to an average rate of occurrence for the primary CV outcome of 3.6 (2.4) %/year [BSR 0.5–11.8] (*comparator*) and 3.0 (1.9)%/year [BSR 0.3–9.1] (*treatment*), respectively (Table 4). The slopes of the equations relating PO rates (y) to diabetes prevalence (x) did not differ according to whether they were derived from PP or SP trials: thus, for PP trials y = 0.0208* x + 0.53 (R^2^ = 0.6369; *p* = 0.0058), whereas y = 0.0267* x +3.76 (R^2^ = 0.1436; *p* = 0.0464) for SP trials.

When comparing PO rates from the comparator arms of studies published prior to 2005 *vs.* those published ≥2005, average PO incidence decreased from 3.7 %/year [<2005] to 2.7 %/year [≥2005] for non-diabetic patients, ie. absolute and relative reductions of 1 % and 28 % (NS). For diabetic patients, the event rate decreased from 5.0 %/year [<2005] to 4.3 %/year [≥2005], ie. absolute and relative reductions of 0.7 % and 14 % (NS).

Among these, 33 trials, totaling 259,151 patients, are described below as *predominantly non-diabetes studies* [12–14, 19–22, 25–29, 31–34, 36–42, 47–66, 68–70, 75, 78–80, 82–90] (Table 1). The mean age was 61.4 (5.5) years [BSR 47.0–75.0], and the proportion of males was 78.6 (17.8) % [BSR 31.4–100]. Among *predominantly non-diabetes studies*, 4 of 33 (12 %) enrolled patients who were in PP at baseline; 9 of 33 (27 %) included mixed populations whose CV risk was either PP or SP; and 20 of 33 (61 %) were clinical trials in SP only. Lipid values at baseline were (mg/dL): 212 (38) [TC]; 129 (36) [LDL-C]; 44 (7) [HDL-C]; 165 (36) [non-HDL-C]; 98 (21) [apoB] and 160 (25) [TG]. In total, these studies have included 63.189 diabetic patients, representing 21.3 % [BSR 2.3 %–44.2 %] of the population studied (Table 1; Table 3). These *predominantly non-diabetes studies* investigated the following interventions over a mean (1SD) duration of 4.3 (1.5) years [BSR: 1.0–7.5 years]: statins (19 trials); fibrates (6 trials); n-3 fatty acids (2 trials); niacin (4 trials); CETP-inhibitor (2 trials); ezetimibe (1 trial); and Lp-PLA2 inhibitor (1 trial) (Table 4).

Amongst *predominantly non-diabetes studies*, we identified 9 diabetes sub-studies (DSS), numbering 12,732 patients, published as *pre-/post-hoc* sub-group analyses of DM patients [14, 29, 32, 38, 49, 52, 54, 86, 90] (Table 1). The mean age was 60.4 (5.3) years [BSR 49.0–65.0], and the proportion of males was 74.9 (12.8) % [BSR 56.2–100]. Within DSS, 2 of 9 (22 %) enrolled patients who were in PP at baseline; 2 of 9 (22 %) included mixed populations whose CV risk was either PP or SP; and 5 of 9 (56 %) were clinical trials in SP only. Lipid values at baseline were (mg/dL): 219 (45) [TC]; 140 (41) [LDL-C]; 41 (5) [HDL-C]; 178 (44) [non-HDL-C]; and 181 (25) [TG] (Table 3). The DSS have investigated the following interventions over a mean (1SD) duration of 4.4 (1.0) years [BSR: 2.8–5.4 years]: statins (7 trials); and fibrates (2 trials) (Table 4).

Fourteen other trials, totaling 71,225 patients, dealt exclusively with DM patients, or included a very-high proportion (>45 %) of DM patients at baseline [11, 15–18, 23, 24, 30, 35, 43–46, 67, 71–74, 76, 77, 81], and are described below as *studies focusing on diabetes* (Table 1). The mean age was 62.6 (8.2) years [BSR 46.0–85.0], and the proportion of males was 63.0 (8.3) % [BSR 42.5–74.4]. Mean diabetes duration was 7.5 (4.9) years [BSR 0–18.0], and HbA1_c_ 7.6 (0.7) % [BSR 6.7–8.6] (Table 3).

Among *studies focusing on diabetes*, 4 of 14 (29 %) enrolled patients who were in PP at baseline; 8 of 14 (57 %) included mixed populations whose CV risk was either PP or SP; and 2 of 14 (14 %) were clinical trials in SP only. Lipid values at baseline were (mg/dL): 200 (19) [TC]; 118 (16) [LDL-C]; 46 (6) [HDL-C]; 154 (19) [non-HDL-C]; and 165 (32) [TG] (Table 3). The *studies focusing on diabetes* investigated the following interventions over a mean (1SD) duration of 4.8 (2.7) years [BSR: 1.8–13.3 years]: statins (5 trials); fibrates (4 trials); n-3 fatty acids and/or traditional Mediterranean diet (3 trials); PPAR-γ agonist (2 trials); and PPAR-α/γ agonist (1 trial) (Table 4).

Among the 33 *predominantly non-diabetic studies*, a total of 14,732 and 12,604 events occurred in the comparator and treatment arms, respectively. On an annual basis, this was equivalent to an average rate of occurrence for the primary CV outcome of 3.8 (2.4) %/year [BSR 0.5–11.8] (*comparator*) and 3.1 (1.8) %/year [BSR 0.3–7.5] (*treatment*), respectively.

Amongst the 9 DSS, a total of 1,469 and 1,119 events occurred in the comparator and treatment arms, respectively. On an annual basis, this was equivalent to an average rate of occurrence for the primary CV outcome of 6.1 (3.0) %/year [BSR 2.1–10.8] (*comparator*) and 4.0 (2.1) %/year [BSR 0.7–7.8] (*treatment*), respectively.

Among the 14 *studies focusing on diabetes*, a total of 3,713 and 3,552 events occurred in the comparator and treatment arms, respectively. On an annual basis, this was equivalent to an average rate of occurrence for the primary CV outcome of 3.3 (2.5) %/year [BSR 1.1–9.6] (*comparator*) and 2.9 (2.4) %/year [BSR 0.8–9.1] (*treatment*), respectively.

**Fig. 1:**
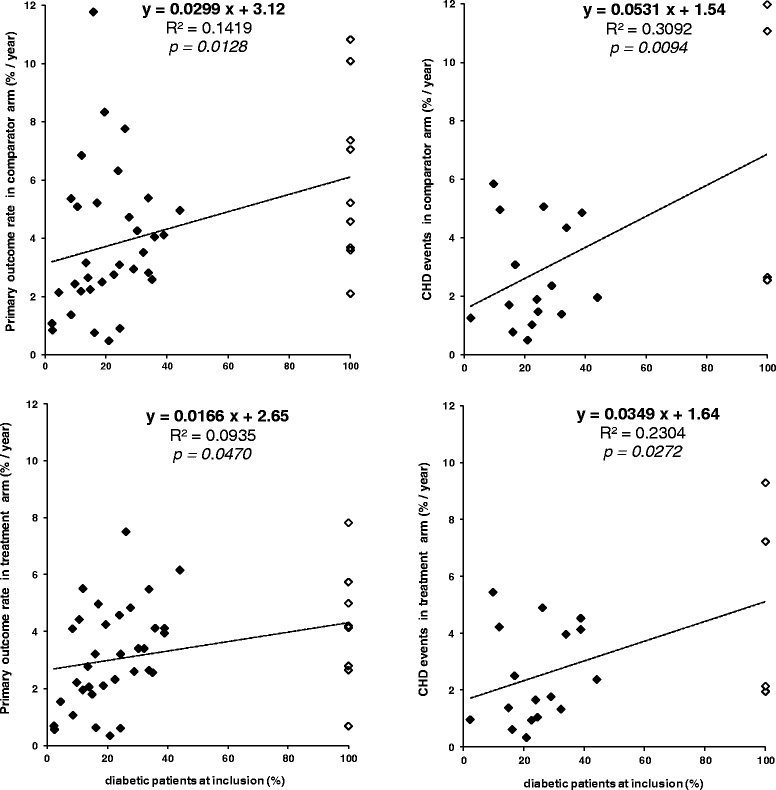
Relationship between proportion of diabetic patients at inclusion (%) and *primary outcome rates* (%/year; *left panels*) or *total coronary heart disease (CHD) events* (%/year; *right panels*) in *comparator arms* (*upper panels*) and in *treatment arms* (*lower panels*) of 33 landmark trials that included a substantial minority of diabetics (ranging from 2 % to 44 %), representing a total of 259,151 patients. The graphs are based on data from the following trials: *4S; AFCAPS/TexCAPS; AIM-HIGH; ALERT; ALLHAT-LLT; Alpha-Omega; ASCOT-LLA; AURORA; BIP; CARE; CDP; dal-OUTCOMES; GISSI-Prevenzione; GREACE; HATS; HHS; HPS-MRC/BHF; HPS2-THRIVE; IDEAL; ILLUMINATE; JELIS; LEADER; LIPID; LIPS; MEGA; PERFORM; Post-CABG; PROSPER; SHARP; STABILITY; TNT; VA Cooperative Study;* and *VA-HIT*. The *open diamonds* represent primary outcome rates and CHD events from the following diabetes substudies: *4S; ASCOT-LLA; AURORA; CARE; GREACE; HHS; HPS-MRC/BHF; TNT; and VA-HIT*. See Table 1 for acronyms definition and trials’ references, and Table 2 and Table 4 for primary outcomes classification and description

In addition to PO rates, which include *de facto* CHD, we also examined CHD rate as a separate outcome [Table 4 and Fig. 1*left panels*]. Rates of CHD were issued for 21 trials and DSS for comparator and treatment arms, and amounted to [%/year]: 11.1 and 7.2 [4S-DSS]; 1.3 and 0.9 [AFCAPS/TexCAPS]; 1.5 and 1.0 [ASCOT-LLA]; 5.1 and 4.9 [AURORA]; 5.8 and 5.4 [BIP]; 12.0 and 9.3 [CARE-DSS]; 4.9 and 4.5 [CDP (clofibrate)]; 4.9 and 4.1 [CDP (niacin)]; 2.4 and 1.7 [HPS - MRC/BHF]; 2.6 and 2.0 [HPS - MRC/BHF-DSS]; 1.4 and 1.3 [HPS2-THRIVE]; 5.0 and 4.2 [IDEAL]; 2.0 and 2.4 [ILLUMINATE]; 0.8 and 0.6 [JELIS]; 3.1 and 2.5 [LEADER]; 0.5 and 0.3 [MEGA]; 1.0 and 0.9 [SHARP]; 4.3 and 4.0 [STABILITY]; 1.7 and 1.4 [TNT]; 2.6 and 2.1 [TNT-DSS]; and 1.9 and 1.7 [VA Cooperative Study] (Fig. 1; *right panels*).

The relationship between proportion of diabetic patients at inclusion and PO or CHD rates was inferred on the basis of the comparator and treatment arms data from the 33 *predominantly non-diabetic studies*, including where appropriate the rates for the corresponding DSS, ie 259,151 patients. Both for PO and CHD, there was a highly significant linear relationship between the proportion of diabetics enrolled and events rates, both in comparator arms (p = 0.0128 [PO] and p = 0.0094 [CHD]; Fig. 1; *upper panels*) and active arms (p = 0.0470 [PO] and p = 0.0272 [CHD]; Fig. 1; *lower panels*). When comparing the slopes of the equations between PO and the proportion of diabetes at baseline in the comparator arm of studies published < 2005 and from 2005 to 2014, they rose from 0.0129 to 0.0162, ie a relative increase of 26 % (*not shown*). Such relationships were more pronounced as regards CHD events, exhibiting steeper gradients than those of PO rates, with slope coefficients higher by a relative 78 % [comparator arms] and 110 % [treatment arms]. Vis-à-vis the comparator arms, the slopes of the relationships between proportions of diabetics and events rates in the treatment arms of the same studies were attenuated, by a relative 45 % [PO rates] and 34 % [CHD events] (Fig. 1; *lower panels*).

Computing occurrence rates of PO and CHD in the *comparator* arms showed that the proportion of diabetics at inclusion predicted PO rates ranging from 3.12 %/year (no diabetic included) to 6.11 %/year (all patients diabetic). Predicted CHD rates depending on baseline diabetes prevalence ranged from 1.54 %/year (no diabetic included) to 6.85 %/year (all patients diabetic). This implies that a cohort exclusively composed of diabetic patients would present a PO rate already increased by an absolute 3 %/year due to the mere fact of being diabetic at baseline. Such an out-of-hand absolute increase in events rate due to the diabetic state would further increase to 5.3 %/year when it comes to the risk of incident CHD (Fig. 1; *upper panels*).

By relating incidence rates of PO and CHD in the *treatment* arms, it appears that the proportion of diabetics at inclusion predicts PO rates ranging from 2.65 %/year (no diabetic included) to 4.31 %/year (all patients diabetic). Predicted CHD rates based on diabetes prevalence ranged from 1.64 %/year (no diabetic included) to 5.13 %/year (all patients diabetic). It follows that a cohort exclusively composed of diabetic patients would present an on-treatment PO rate increased by an absolute 1.7 %/year solely due to the presence of DM at baseline. Such an absolute increase in events rate due to diabetes would further increase to 3.5 %/year for incident CHD risk (Fig. 1; *lower panels*).

The comparison of these equations linking the proportion of diabetics and outcome rates in comparator vs. treatment arms allows for determining whether being diabetic (apart from the observation that it increases the absolute rate of occurrence of CV events) is associated with an idiosyncratic on-treatment clinical response. As for PO and CHD, diabetic patients were characterized by a clinical response that was better than that calculated for a non-diabetic population that would have been subject to the same therapeutic interventions. Thus, residual CV risk persisting after treatment was further reduced in case of diabetes, in a relative proportion of 14.4 % [PO] and 31.2 % [CHD], respectively (Fig. 1; *upper and lower panels*).

## Discussion

This meta-analysis shows that the presence of diabetics in a lipid-modifying trial is a determinant of CV events rate, the impact of which can be accurately assessed once known the proportion of diabetics enrolled, regardless of the CV risk category at baseline. Thus, the linear equations derived from this meta-analysis can be used to determine the absolute and relative enhancement of CV risk related to the inclusion of diabetics in a trial. Conversely, these algorithms can be used to estimate the proportion of diabetics to be included when designing a prospective study, in order to achieve a given number of CV events.

Major guidelines recognize a higher risk of CHD in DM patients, even in situations of primary prevention, as compared to non-diabetic subjects. The events rates in the comparator arms of randomized controlled trials and the meta-analyses of key statin trials show that CHD risk from hypercholesterolemia in non-diabetic patients is proportional to baseline LDL-C level. This is also the case for type 2 DM patients, with the additional aggravating fact that this linear relationship was shifted upward compared to non-diabetics. This underlies current recommendations for effective lowering of LDL-C as the major modifiable lipid risk factor for CHD in diabetic patients.

It should be noted that mean PO rate in *studies focusing on diabetes* was considerably lower (-46 %) than the risk that would be determined for diabetics if included, as a subgroup, in a clinical trial not focusing on diabetes. This follows from the fact that *studies focusing on diabetes* had a lower CV risk at inclusion, as well as lesser PO or CHD events during the study. As a result, the impact of DM on CV events must be qualified according to whether it is evaluated from diabetic subgroups of cohorts followed in cardiology (mostly in a macrovascular setting), or whether it is obtained in patients from clinical trials focusing on nutrition or diabetes (usually dealing with glycemic control or microvascular risk reduction). In addition, variation in residual risk related to T2DM in key trials may result from inhomogeneity in inclusion criteria; varying baseline CV risk; individual differences in diabetes duration or severity; and heterogeneous RFs exposure among diabetics.

As opposed to what occurs in microvessels, and unlike a widely held view about it, residual risk targeting large vessels is related to a limited extent only by hyperglycaemia in (pre)diabetes states. Rather, the accrued macrovascular risk is associated with the common form of T2DM (that is to say the one that expresses a MetS phenotype, including insulin resistance and hyperinsulinemia). The common pathogenic factors underlying the observed association between hyperglycemia and CHD are involved either (*i*) at the onset of diabetes (promoting B-cell decompensation or altering one or two variable(s) of the hyperbolic product between insulin secretion and insulin sensitivity), and/or (*ii*) because they embody cardiometabolic comorbidities that increase the macrovascular risk regardless of glucose levels.

It should be noted that the slopes of the relationships between CV events and percentage of included diabetics were less marked when it came to comparing PO vs. CHD events rates, both in comparator and treatment arms, on one hand, or when it came to comparing PO or CHD events rates in treated arms vs. comparator arms, on the other hand. These observations suggest (*i*) that the presence of diabetes at baseline has less adverse effect on the occurrence of certain constituents of the PO, such as all-cause deaths or coronary revascularization; and (*ii*) that diabetic patients derive more benefits from the different treatment approaches studied than non-diabetic patients as regards the occurrence of macrovascular events [91]. In this meta-analysis, we have not distinguished between studies on the basis of pharmacological or nutritional interventions, since we based our findings on patients from comparator arms, usually receiving a *placebo* or standard care. When comparing less recent (published <2005) and more contemporary studies (published ≥2005), a decrease in absolute and relative events rates was observed (-28 % and -1 % respectively), suggestive of a reduction in exposure to CV RFs over time and/or of improved overall CV management. Such changes were however not significant and further, diabetic patients benefited less from this trend, reducing the absolute and relative rates by only -14 % and -0.7 %. It seemed therefore appropriate to include all studies in this analysis regardless of publication year.

It is noteworthy that the increased risk of CV events due to the presence of a subgroup of diabetics had a pretty similar slope, whatever the CV risk category at baseline. It follows that the excess CV risk associated with the inclusion of people with diabetes in a lipid-modifying trial is relatively independent of study design, expanding the applicability of equations derived from this meta-analysis. There exists a positive relationship between biomarkers and occurrence of CV events [92]; our meta-analysis suggests that documenting the frequency of enlisted T2DM patients can also be used as surrogate biomarker predicting a non-modifiable component of residual CV risk. Considering that our analysis focused on populations enrolled in the comparator arms of mostly LMT studies, it would be interesting to determine the impact on residual risk arising from enlistment of diabetics in clinical trials testing several interventions in primary care [93].

This study has several limitations. Firstly, the risk estimates attributed to DM were not adjusted for age or other CV RFs comorbid to T2DM and, as in all systematic collection of published data, there is always a potential bias related to publications [94]. Secondly, the adequacy of these equations to predict CV outcomes has not been independently validated in a prospective context. Thirdly, for reasons related to the design and reporting of individual studies, it was not feasible to derive specific equations applicable to T1DM *vs.* T2DM subgroups, or to newly-diagnosed vs. long-standing T2DM patients [95]. We were not able to analyze the potential influence of glycaemic control in diabetic subgroups at baseline, due to the low reporting rate of HbA_1c_ values [96]. Finally, we did not examine, for reasons of brevity, the relationship between diabetes prevalence and non-CHD outcomes, such as HF, which will require dedicated meta-analyses [97].

## Conclusion

This study attempted to quantify the impact of diabetes on the occurrence of CV events during a lipid-modifying trial, based on the proportion of known diabetics included. The component of absolute and relative residual CV risk associated with diabetes can be measured from linear equations relating diabetes prevalence to primary outcomes or CHD rates. Such calculations may help clinical study designers when selecting inclusion criteria; cohort size; and planned diabetics’ enrollment, so as to achieve sufficient CV events over time.

## Abbreviations

apoB: apolipoprotein B_100_
BSR: Between-study range
CETP: Cholesteryl ester transfer protein
CHD: Coronary heart disease
CV: Cardiovascular
DM: Diabetes mellitus
DSS: Diabetes substudy
HbA_1c_: glycated hemoglobin
HDL: High-density lipoprotein
HDL-C: High-density lipoprotein cholesterol
LDL: Low-density lipoproteins
LDL-C: Low-density lipoprotein cholesterol
Lp-PLA2: Lipoprotein-associated phospholipase A2
non-HDL-C: non-high-density lipoprotein cholesterol
NS: Non-significant
PO: Primary outcome
PP: Primary prevention
PPAR: Peroxisome proliferator-activated receptor
RF: Risk factor
SD: Standard deviation
SP: Secondary prevention
T1DM: Type 1 diabetes mellitus
T2DM: Type 2 diabetes mellitus
TC: Total cholesterol
TG: Triglycerides (triacylglycerols)
